# Endurance Exercise Accelerates Myocardial Tissue Oxygenation Recovery and Reduces Ischemia Reperfusion Injury in Mice

**DOI:** 10.1371/journal.pone.0114205

**Published:** 2014-12-04

**Authors:** Yuanjing Li, Ming Cai, Li Cao, Xing Qin, Tiantian Zheng, Xiaohua Xu, Taylor M. Sandvick, Kirk Hutchinson, Loren E. Wold, Keli Hu, Qinghua Sun, D. Paul Thomas, Jun Ren, Guanglong He

**Affiliations:** 1 School of Pharmacy, University of Wyoming, Laramie, Wyoming, United States of America; 2 Department of Kinesiology & Health, University of Wyoming, Laramie, Wyoming, United States of America; 3 Department of Physiology and Cell Biology, The Ohio State University, Columbus, Ohio, United States of America; 4 Division of Environmental Health Sciences, The Ohio State University, Columbus, Ohio, United States of America; 5 Division of Pharmacology, The Ohio State University, Columbus, Ohio, United States of America; 6 Department of Physiology, University of Arizona, Tucson, Arizona, United States of America; 7 Department of Cardiology, The First Affiliated Hospital of Chongqing Medical University, Chongqing, People’s Republic of China; 8 Endocrinology and Breast Surgery, The First Affiliated Hospital of Chongqing Medical University, Chongqing, People’s Republic of China; 9 Department of Pharmacology, Soochow University, Soochow, Jiangsu, People’s Republic of China; 10 Department of Cardiology, Fourth Military Medical University, Xi’an, Shaanxi, People’s Republic of China; Indiana University School of Medicine, United States of America

## Abstract

Exercise training offers cardioprotection against ischemia and reperfusion (I/R) injury. However, few essential signals have been identified to underscore the protection from injury. In the present study, we hypothesized that exercise-induced acceleration of myocardial tissue oxygenation recovery contributes to this protection. C57BL/6 mice (4 weeks old) were trained on treadmills for 45 min/day at a treading rate of 15 m/min for 8 weeks. At the end of 8-week exercise training, mice underwent 30-min left anterior descending coronary artery occlusion followed by 60-min or 24-h reperfusion. Electron paramagnetic resonance oximetry was performed to measure myocardial tissue oxygenation. Western immunoblotting analyses, gene transfection, and myography were examined. The oximetry study demonstrated that exercise markedly shortened myocardial tissue oxygenation recovery time following reperfusion. Exercise training up-regulated Kir6.1 protein expression (a subunit of ATP-sensitive K^+^ channel on vascular smooth muscle cells, VSMC sarc-K_ATP_) and protected the heart from I/R injury. In vivo gene transfer of dominant negative Kir6.1AAA prolonged the recovery time and enlarged infarct size. In addition, transfection of Kir6.1AAA increased the stiffness and reduced the relaxation capacity in the vasculature. Together, our study demonstrated that exercise training up-regulated Kir6.1, improved tissue oxygenation recovery, and protected the heart against I/R injury. This exercise-induced cardioprotective mechanism may provide a potential therapeutic intervention targeting VSMC sarc-K_ATP_ channels and reperfusion recovery.

## Introduction

Ischemic heart disease is a major cause of morbidity and mortality in the United States and is often associated with increased prevalence of obesity and type 2 diabetes due to habitual physical inactivity and excess caloric intake [1]–[4]. Exercise is an effective lifestyle intervention to prevent cardiovascular disease [5] and displays beneficial effects for atherosclerosis [6], [7], mitochondrial biogenesis [3], blood flow [8], and myocardial ischemia and reperfusion (I/R) injury [9]–[11]. However, the essential mechanisms responsible for exercise-induced cardioprotection remain largely elusive [5].

Following ischemia, rapid opening of the occluded coronary arteries and prompt reperfusion of the myocardial tissue are critical to the ultimate survival of the myocardium at risk. However, clinically, percutaneous coronary intervention following acute myocardial infarction is often accompanied by compromised reperfusion with adverse post-ischemic remodeling and poor prognostic outcome [12]–[14]. Therefore, interventions aiming at improving reperfusion recovery following ischemia are extremely critical to the salvage of the ischemic myocardium.

Membrane ATP-dependent K^+^ channels (K_ATP_) in cardiomyocytes (with Kir6.2 subunit) and smooth muscle cells (VSM) (with Kir6.1 subunit) play important roles in the homeostasis of myocardial function. Exercise-induced up-regulation of Kir6.2 in cardiomyocytes confers cardioprotection [15]. Nonetheless, the precise role of exercise on Kir6.1 regulation in the vasculature and heart remains unclear. Given the specificity issues of K_ATP_ channel openers and inhibitors, it is pertinent to dissect isoform-specific roles of vascular versus cardiomyocyte K_ATP_ channels for targeted pharmacological interventions.

Opening of the VSMC sarc-K_ATP_ channels during ischemia via accumulation of ADP hyperpolarizes VSMC membrane potential and relaxes vascular tone [16], [17]. In addition to the response of specific VSMC sarc-K_ATP_ channel openers and inhibitors, Kir6.1 expression can be specifically up-regulated by certain circulating hormones such as urocortins [18], [19]. Determining whether exercise regulates Kir6.1 and exerts cardioprotective effects may shed light on future specific pharmacological or molecular interventions.

Using a mouse model of treadmill exercise training followed by coronary ligation and reperfusion, the current study was designed to elucidate a novel mechanism on the cardioprotective effects of exercise through shortening reperfusion recovery time and prompt restoration of tissue oxygenation and blood flow following ischemia.

## Materials and Methods

### 1. Animals

Male C57BL/6 mice were obtained from Jackson Laboratory (Bar Harbor, ME) at 3 weeks of age. Mice were housed in the animal care facility on the ground floor of The Davis Heart and Lung Research Institute, The Ohio State University, and School of Pharmacy, University of Wyoming College of Health Sciences for one week before the exercise training started. These facilities are administered by the University Laboratory Animal Resources Centers (ULAR) of The Ohio State University and University of Wyoming and provide veterinary supervision of care, and ULARs are accredited by the American Association for Accreditation of Laboratory Animal Care. All animal studies were approved by The Ohio State University’s and University of Wyoming’s Institutional Animal Care and Use Committee (IACUC) and the investigation conforms to the federal guidelines for the humane and appropriate care of laboratory animals, Federal Law (89–544, 91–579) and all NIH regulations.

### 2. Treadmill exercise training regimen

Our exercise protocol was developed according to an established exercise training regimen from our lab as well as others [6], [20], [21]. Starting at 4 weeks of age, male C57BL/6 mice were trained for 8 weeks on a treadmill (EX, Columbus Instruments) for 45 min/day for 7 days/week at 15 m/min in the late afternoon during the regressive phase. The mice were acclimated to the treadmill for 3 days before running: on the unmoving treadmill for 10 min followed by 5 m/min for 10 min and 15 m/min for 10 min for each day. All the measurements were performed the day following the 8-week exercise training.

### 3. Kir6.1AAA packaging and delivery

A dominant negative gene plasmid Kir6.1AAA (with the pore-forming region Gly-Phe-Gly amino acid residues mutated to Ala-Ala-Ala) was sub-cloned to an adenovirus vector tagged with green fluorescence probe (GFP). The viral construct of Kir6.1AAA-GFP (Ad-Kir6.1AAA-GFP) was obtained from Vector BioLabs (Philadelphia, PA) at a concentration of ∼1.4×10^11^ PFU/mL. The mice were transfected with Ad-Kir6.1AAA-GFP and Ad-GFP for 7 days using i.v. injection of ∼1.4×10^9^ PFU/mouse as per the manufacturer’s instructions.

### 4. Acute ischemia and reperfusion model

Following 8 weeks of exercise training, mice were anesthetized with an i.p. injection of ketamine (55 mg/kg)/xylazine (15 mg/kg) [22]. The trachea was exposed by an incision under the neck, a cannula was inserted into the trachea and the mice were artificially ventilated with 1% isoflurane in 21% oxygen. Body temperature was controlled at 37±0.5°C by placing the animals on a thermal control pad with temperature adjusted based on measurements of core body temperature using a rectal thermistor. As an approximation, mice were ventilated with a tidal volume of 200 to 250 µL at a rate of 90/min.

Access to the heart was through an intercostal incision. The pectoralis muscle was retracted medially and the rectus laterally using a pair of tissue forceps while performing blunt dissection. The chest wall was punctured between the 4^th^ and 5^th^ ribs. Retractors were used to create a working area and a suitable view of the heart. A 7-0 silk suture was inserted into the heart medially below the atrium around the left anterior descending (LAD) artery. Regional ischemia was induced by LAD ligation over a piece of PE-10 tubing at a point 1 to 2 mm below the left auricle and confirmed by discoloration of the myocardium and changes in cardiac rhythm. Reperfusion was achieved by release of the ligature and confirmed visually. Mice were subjected to 30 min LAD occlusion followed by 60 min and 24 h reperfusion for acute phase oximetry and infarct size measurements.

### 5. In vivo measurement of myocardial tissue oxygenation

For measuring myocardial tissue oxygenation, ∼10 µg of lithium phthalocyanine (LiPc, the oxygen-sensitive probe with a sensitivity of 5.9 mG/mmHg) was implanted in the mid-myocardium of the area at risk (AAR) using a 25-gauge needle following exposure of the heart, as described previously [23]. The mouse was then moved to an L-band electron paramagnetic resonance (EPR) spectrometer (Magnettech GmbH, Germany) and EPR spectra were collected with the following parameters: frequency 1.1 GHz, microwave power 16 mW, modulation field amplitude 45 mG.

### 6. Infarct size measurement

After 30 min occlusion of the LAD, the ligature was released and left in place. The chest was then sutured in layers to assure recovery. At 24 h reperfusion, mice were re-anesthetized and hearts excised and cannulated with a 23-gauge needle through the ascending aorta. About 3 to 4 mL of 1.0% triphenyl tetrazolium chloride (TTC) in phosphate buffer (pH 7.4, 37°C) was injected through the cannula. The LAD was then re-occluded by tightening of the suture in the myocardium and the hearts were perfused with 2–3 mL of 5% Evans blue to demarcate the AAR. The hearts were then weighed, frozen, and cut into five transverse slices, each at ∼1 mm thickness. Each slice was photographed from both sides with a digital camera on a dissecting microscope. The sections were contoured with a planimeter. Sizes of the non-ischemic area, AAR, and infarct area were calculated as percentages of the total left ventricle (LV) area and AAR.

### 7. Echocardiography

Coronary blood flow (CBF) was measured using a high-frequency, high-resolution ultrasound unit (Vevo 2100, Visual Sonics, Toronto, Canada). The LAD diameter and flow were measured under a modified four chamber view with guidance using color Doppler. Mice were anesthetized using 5% isoflurane. Following sedation, isoflurane was reduced to 1% to determine baseline coronary flow, and then increased to 3% to measure maximal coronary flow. CBF was calculated using the following equation: 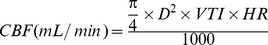 (where D is the internal coronary diameter (in mm) measured in B-mode ultrasound images, VTI is the velocity-time-integral (in mm), or area under the curve of the Doppler blood flow velocity tracing, and HR is heart rate [24].

### 8. Western immunoblotting analyses

To measure protein expression of Kir6.1, frozen tissue from the AAR was thawed, finely minced and homogenized. The tissue homogenate was boiled in NuPAGE LDS sample buffer (Invitrogen) for 10 min. Protein homogenates were subjected to electrophoresis on NuPAGE Novex 4–12% Bis-Tris gels (Invitrogen) and transferred onto nitrocellulose membranes (Amersham Biosciences). After blocking with 5% non-fat milk in Tween-20- and Tris-buffered saline (TTBS) for 1 h at room temperature, the membranes were incubated with goat polyclonal antibodies against Kir6.1 and glyceraldehyde-3-phosphate dehydrogenase (GAPDH) (1∶1000, Santa Cruz Biotechnology). After incubation, the membranes were washed with TTBS and exposed to the antibodies conjugated with horseradish peroxidase for 1 h at room temperature. Proteins were detected using chemiluminescence Western immunoblotting detection reagents (Amersham Biosciences). Densitometric analyses of the immunoblots were performed using an Alpha Imager 3300 system (Alpha Innotech, San Leandro, CA).

### 9. Myography

Thoracic aortic rings (2 mm each) excised from the mice were mounted in individual organ chambers that were filled with physiological salt solution, as described previously [20]. The rings were subjected to graded doses of vasoconstrictor phenylephrine (PE: 10^−9^ to 10^−5^ M). After a stable contraction plateau was reached (PE: 10^−5^ M), the rings were exposed to the endothelium-dependent vasodilator acetycholine (ACh: 10^−9^ to 10^−5^ M), the K_ATP_ channel opener diazoxide (Diaz: 1×10^−5^ to 11×10^−5^ M), and dose-response force was measured. To inhibit the K_ATP_ channel, vessels were pre-treated with glibenclamide (Glib: 10^−4^ M) for 30 min followed by PE (10^−5^ M) and Diaz exposure (1×10^−5^ to 11×10^−5^ M).

### 10. Statistical Analysis

Two-way ANOVA was used for data analyses of Po_2_ and myography. A one-way ANOVA was used for protein expression; these were followed by Student-Newman-Keuls multiple-comparison tests among the groups. Generally, the number of animals was 4 to 6/group, except for the Po_2_ measurements where N = 7. Data are represented as means ± SEM. A value of p<0.05 was considered statistically significant.

## Results

### 1. The effect of exercise on body weight and heart/body weight ratio

At the end of the 8-week endurance exercise training, body weight and heart weight were measured. As shown in **Table 1**, the average body weight of the exercised mice was 20.4±0.9 g, which was significantly lower than the non-exercised mice (24.0±0.5 g). However, the heart-to-body weight ratio displayed no significant difference between the two groups, indicating absence of hypertrophy with our exercise protocol.

**Table 1:** The effect of 8-week endurance exercise on heart and body weight.

	Body Weight (g)	Heart/body (mg/g)
Control	24.0±0.5 (5)	5.3±0.9 (5)
Exercise	20.4±0.9 (5)*	4.8±0.3 (5)

Data are expressed as mean ± SEM (n), *p<0.05 vs. control.

### 2. Exercise training shortened myocardial tissue oxygenation recovery time

**Figure 1:**
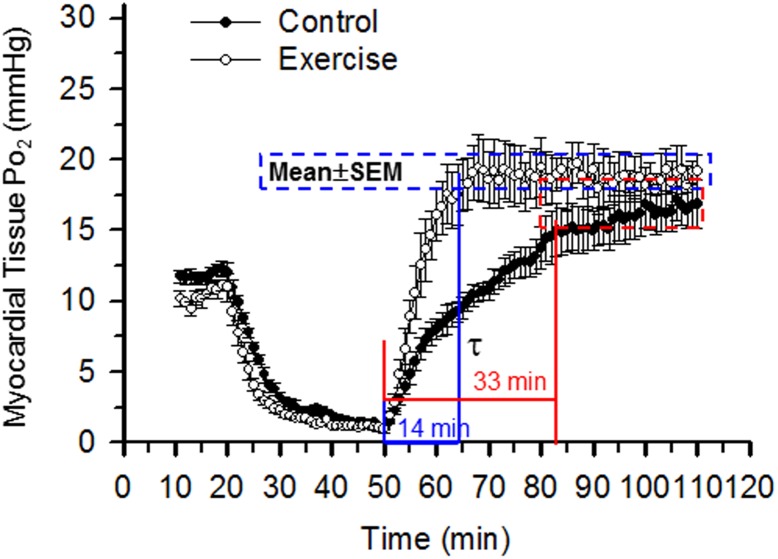
Myocardial tissue oxygenation during ischemia and reperfusion. In vivo EPR oximetry was performed to measure myocardial tissue oxygenation (Po_2_) before (0–20 min), during (20–50 min), and after (50–110 min) ischemia and reperfusion (N = 5/group). Myocardial tissue oxygenation recovery time τ was measured from the start of reperfusion to plateau. As shown, endurance exercise dramatically shortened τ from 33 (control) to 14 min without significant changes in oxygenation levels at baseline or after reperfusion plateaued.

Next, we used EPR oximetry to monitor in vivo myocardial tissue oxygenation in the hearts of the exercised and control mice as described previously [22], [25], [26]. As shown in **Figure 1**, exercise training markedly shortened myocardial tissue oxygenation recovery time τ (defined as from the start of reperfusion to reaching oxygenation plateau) from 33 min to 14 min. Interestingly exercise did not significantly affect oxygenation levels before ischemia or after the plateau.

### 3. Exercise training had no effect on steady state (basal) coronary blood flow

**Figure 2:**
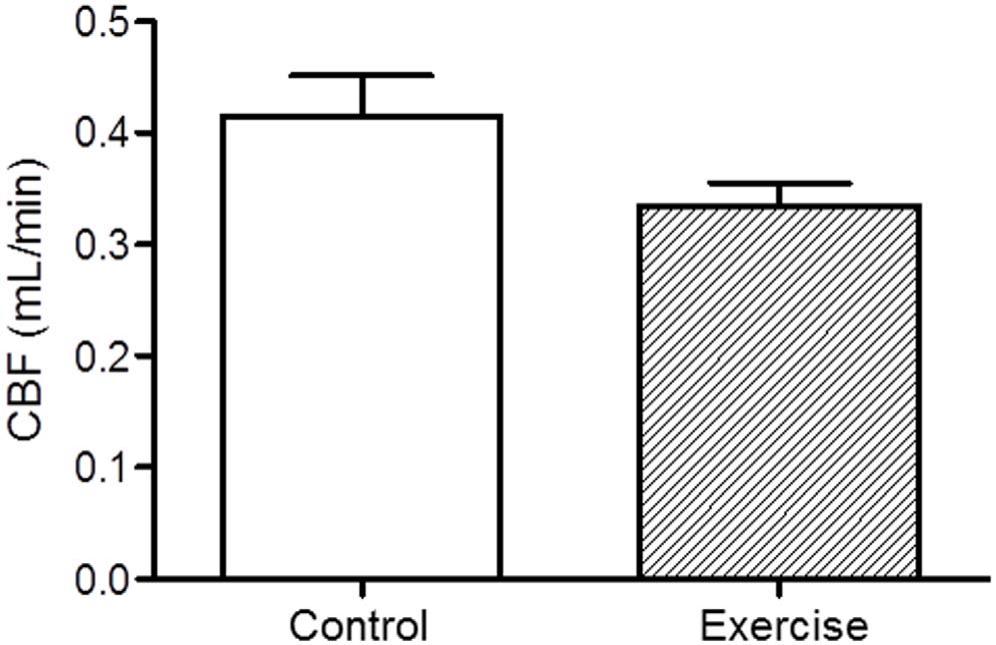
Steady state coronary blood flow. Coronary blood flow (CBF) was measured using echocardiography. Briefly, color flow Doppler was utilized to locate the left anterior descending coronary artery (LAD) and the pulsed wave Doppler sample volume was placed in line with the greatest flow. A modified apical 4-chamber view was used to obtain coronary flow velocities. As shown, there was no significant difference in CBF between the exercised and control groups (N = 5/group).

To determine whether exercise affects steady state myocardial blood perfusion, basal coronary blood flow was measured using echocardiography at the end of the 8-week exercise training. As shown in **Figure 2**, there was no significant difference in steady state coronary blood flow between the exercised and control groups (0.34±0.02 vs. 0.41±0.04 mL/min in the control hearts, N = 5/group, p = NS), which was consistent with the similar tissue oxygenation levels before ischemia or after the plateau, as shown in **Figure 1**.

### 4. Exercise training up-regulated Kir6.1

**Figure 3:**
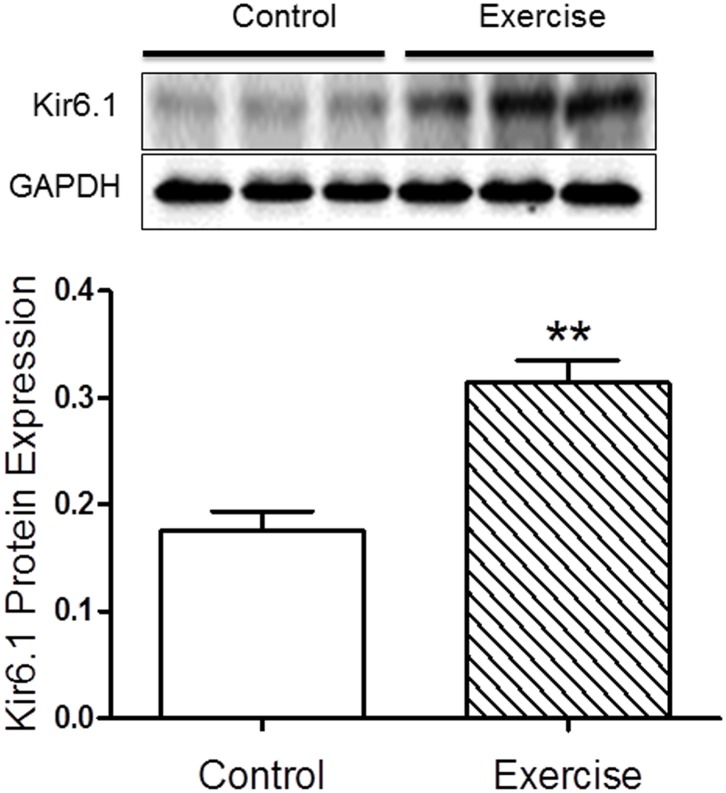
The effect of endurance exercise on Kir6.1 protein expression. Western immunoblotting analyses of VSMC sarc-K_ATP_ channel subunit Kir6.1 were performed on the hearts of the control and exercised mice with GAPDH as the reference. As shown, protein expression levels of Kir6.1 were significantly increased following 8 weeks of endurance exercise (N = 5/group, **p<0.01).

Although a number of signaling pathways were identified for exercise-induced cardioprotection, only few were essential [5], [27], [28]. To date, there are no prior studies on the effect of exercise-induced regulation of VSMC sarc-K_ATP_ channels and myocardial tissue oxygenation recovery following ischemia. To determine whether exercise up-regulates VSMC sarc-K_ATP_, Western blot analyses were performed using a Kir6.1 goat polyclonal antibody to measure the expression of Kir6.1, the pore-forming subunit of the VSMC sarc-K_ATP_ [16]. As shown in **Figure 3**, protein expression of Kir6.1 was significantly up-regulated (∼1.8 fold) following 8 weeks of exercise training.

### 5. Kir6.1AAA gene transfection and vascular activity

To determine how Kir6.1 affects vascular activity, we used gene transfer technique to manipulate the function of VSMC sarc-K_ATP_ channels. Non-exercised control mice were transfected with Ad-Kir6.1AAA-GFP or Ad-GFP viral construct for 7 days as described in the **Materials and Methods** section. Following 7 days of transfection, gross body weight and wet heart weight were similar between the Ad-Kir6.1AAA-GFP-transfected and the control Ad-GFP group. By examining the ECG signal under anesthesia (55 mg/kg ketamine plus 15 mg/kg xylazine), Ad-Kir6.1AAA-GFP transfected mice had a heart rate of 587±17 bpm which was similar to that in the Ad-GFP control group of 586±8 bpm. Further, there was no sign of S-T segment variation and related vasospasm as reported in a Kir6.1 knockout model [16], [17]. Mean arterial blood pressure was measured in conscious mice using the tail-cuff method [29], and there were no significant difference between the two groups (116±6 mmHg vs. 114±8 mmHg in the control).

**Figure 4:**
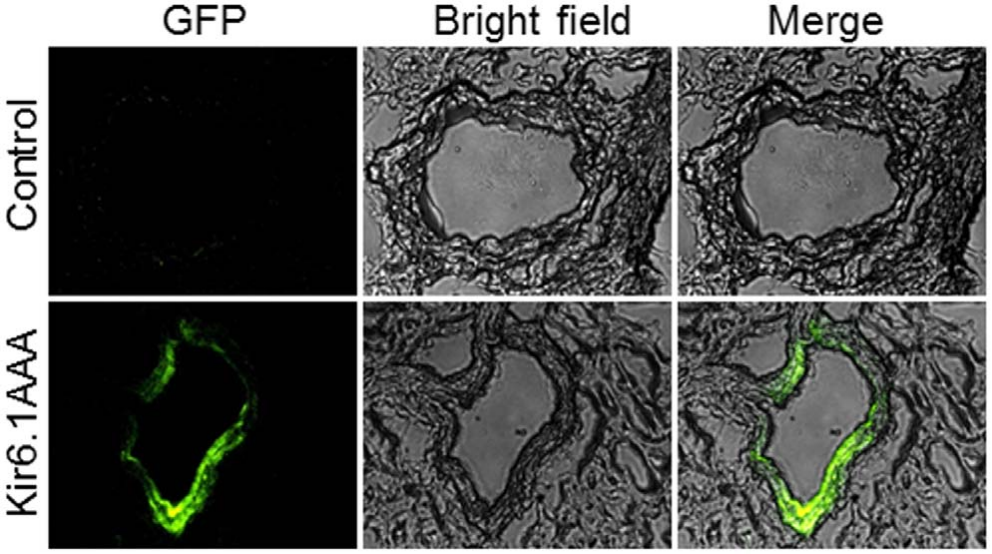
Coronary artery transfection with Kir6.1AAA. Fluorescence microscopy was performed on heart sections containing coronary arteries. Sections were from Control (row 1) and Ad-Kir6.1AAA-GFP transfected (row 2) hearts. Column 1 shows GFP images, column 2 shows the respective bright field images of the vessels, and column 3 shows the merged images. Magnification is 400X. Green fluorescence representing the expression of Kir6.1AAA is clearly evident in the coronary vessel section, indicating successful transfection with Kir6.1AAA.

To determine whether Kir6.1AAA was transfected into the coronary vasculature, Ad-Kir6.1AAA-GFP transfected mouse hearts were excised, frozen in liquid nitrogen-cooled OCT, sectioned, and fixed in −20°C acetone. Then, heart slices were visualized using a fluorescence microscope (Axioskop 40, Zeiss). As shown in **Figure 4**, green fluorescence was predominantly present in the coronary artery (second row) with no visible fluorescence signal in the control hearts (first row).

**Figure 5:**
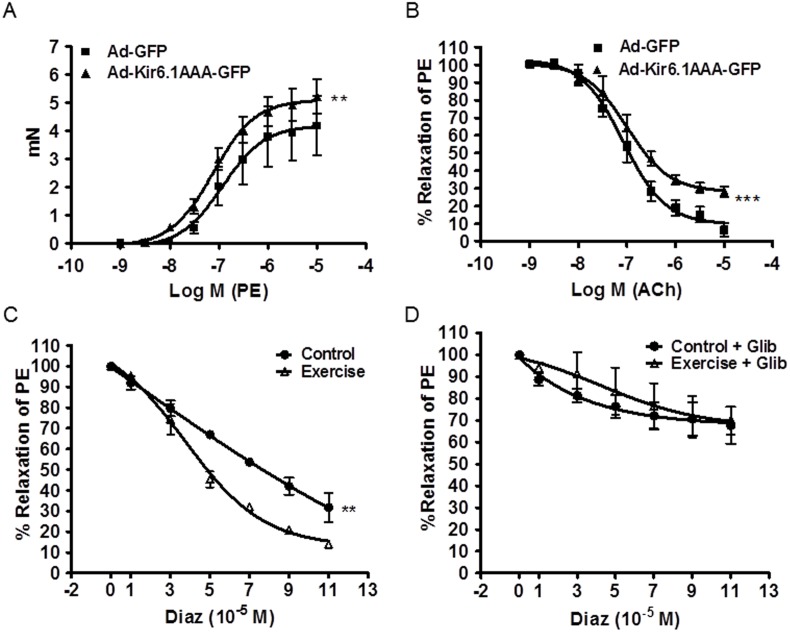
Myography of vasomotor tone in aortic rings. Vasomotor tone changes from control mice transfected with Ad-Kir6.1AAA-GFP and Ad-GFP in response to A: phenylephrine (PE) and B: acetylcholine (ACh); Vasomotor tone changes from the exercised and control mice in response to C: diazoxide (Diaz) and D: glibenclamide pre-treatment and Diaz. **p<0.01, ***p<0.001, N = 5/group.

To determine vascular activity following Ad-Kir6.1AAA-GFP transfection, thoracic aortic rings from the transfected mice were excised and vasomotor tone was evaluated using myography as described previously [20]. As shown in **Figure 5A**, the constriction response to PE in Ad-Kir6.1AAA-GFP transfected mice was significantly higher than that in the Ad-GFP control mice, indicating an enhanced vasoconstriction due to inhibition of the endogenous VSMC K_ATP_ channel function by dominant negative Kir6.1AAA transfection. As shown in **Figure 5B**, relaxation response to the endothelium-dependent vasodilator ACh in Ad-Kir6.1AAA-GFP transfected mice was significantly lower than that in the Ad-GFP control mice, indicating an impaired vessel relaxation due to Kir6.1AAA transfection. As shown in **Figure 5C**, relaxation response to the K_ATP_ channel opener Diaz was significantly increased in the exercised mice compared to that of the control group. The exercise-enhanced relaxation was completely blunted by Glib, a K_ATP_ channel inhibitor, as shown in **Figure 5D**. These results clearly demonstrated that exercise-induced up-regulation of Kir6.1 is responsible for the improved vascular activity.

### 6. Kir6.1AAA transfection prolonged myocardial tissue oxygenation recovery

**Figure 6:**
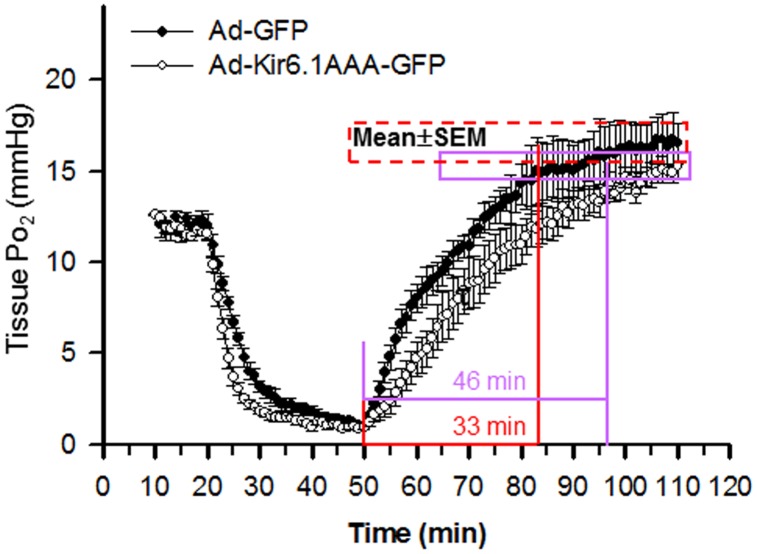
Myocardial tissue oxygenation during ischemia and reperfusion in Kir6.1AAA-transfected mouse hearts. In vivo EPR oximetry was performed to measure tissue Po_2_ in the Ad-GFP and Ad-Kir6.1AAA-GFP transfected hearts before (0–20 min), during (20–50 min), and after (50–110 min) 30 min ischemia and 60 min reperfusion (N = 5/group). As shown, Kir6.1 AAA gene transfer markedly prolonged myocardial tissue oxygenation recovery time τ from 33 to 46 min.

Seven days following transfection, the Ad-Kir6.1AAA-GFP transfected control mice were subjected to LAD occlusion followed by reperfusion. In vivo EPR oximetry was performed before, during, and after ischemia. As shown in **Figure 6**, Kir6.1AAA transfection markedly prolonged oxygenation recovery time τ from 33 to 46 min without significant changes to the oxygenation levels before ischemia or after plateau.

### 7. Exercise protected the heart against I/R injury

**Figure 7:**
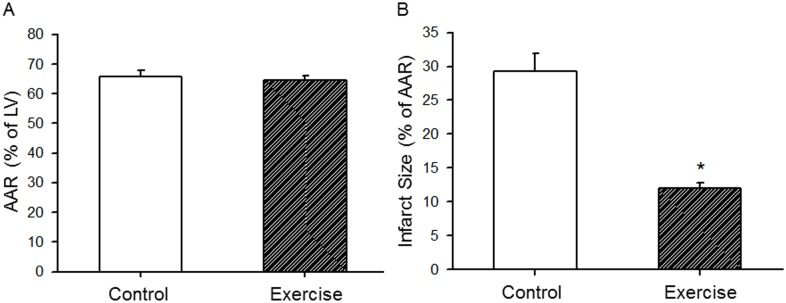
The effect of endurance exercise training on myocardial I/R injury. Following 8 weeks of endurance exercise, mice were subjected to LAD occlusion followed by 24 h reperfusion. Area at risk (AAR) and infarct size were measured with Evans blue and TTC staining. A, there was no significant difference in the AAR (65.7±2.3 vs. 64.6±1.5%, N = 5/group, p = NS). B, infarct size was significantly smaller in the exercised group than that in the control group (29.2±2.7 vs. 12.0±0.8%, N = 5/group, *p<0.01).

Using the regional I/R model [22], [25], [26], we observed exercise-induced cardioprotection. Briefly, after 8 weeks of exercise training, mouse hearts were subjected to 30 min LAD occlusion and 24 h reperfusion. Hearts were then excised and stained with Evans blue and TTC as described in the **Methods** section. As shown in **Figure 7**, there was no significant difference in AAR between the control and exercised mice (65.7±2.3 vs. 64.6±1.5%, N = 5/group, p = NS). However, infarct size in the exercised group was significantly smaller than that in the control group (12.0±0.8 vs. 29.2±2.7%, N = 5/group, *p<0.05).

### 8. Kir6.1AAA transfection exacerbated I/R injury

**Figure 8:**
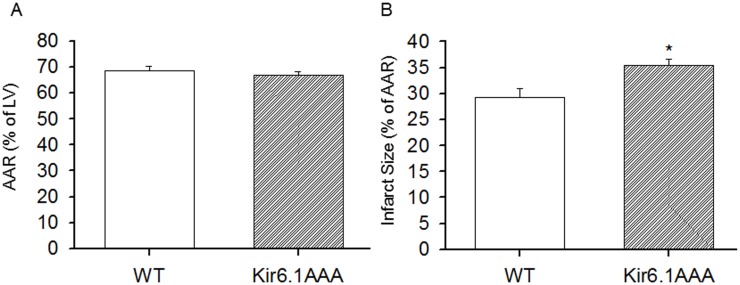
The effect of Kir6.1AAA transfection on myocardial I/R injury in non-exercised mice. After 1 week of Ad-Kir6.1AAA-GFP transfection, control mice (WT) were subjected to LAD occlusion followed by 24 h reperfusion. AAR (A) and infarct size (B) were measured with Evans blue and TTC staining. As shown, there was no significant difference in the AAR, but infarct size was increased ∼21% in the transfected group. N = 7/group, *p<0.05.

**Figure 9:**
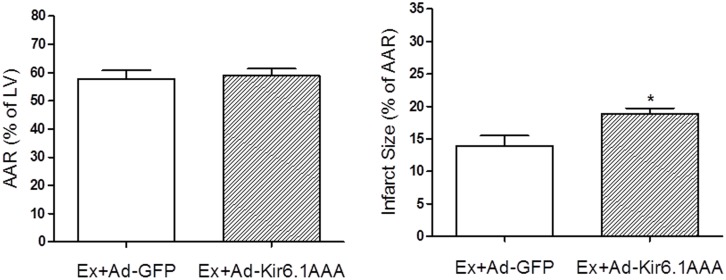
The effect of Kir6.1AAA transfection on myocardial I/R injury in the exercised mice. After 1 week of Ad-Kir6.1AAA-GFP transfection, the exercised mice were subjected to LAD occlusion followed by 24 h reperfusion. AAR (A) and infarct size (B) were measured with Evans blue and TTC staining. As shown, there was no significant difference in the AAR, but infarct size was increased significantly (∼36%) in the Ad-Kir6.1AAA-GFP group compared to that of the Ad-GFP transfected mice. N = 5/group, *p<0.05.

In a separate group of Kir6.1AAA-transfected control mice, Evans blue and TTC staining were performed 24 h following reperfusion to determine the effect of Kir6.1 mutation on I/R injury. As shown in **Figure 8**, there was no significant difference in AAR between the WT control and the Kir6.1AAA-transfected mice, whereas the Kir6.1AAA-transfected mice showed a significant increase in the infarct size (by ∼21%). Similarly, as shown in **Figure 9**, transfection of Kir6.1AAA in the exercised mice significantly increased the infarct size and blunted the protection. These results demonstrated that down-regulation of functional VSMC Kir6.1 prolonged oxygenation recovery time and exacerbated I/R injury.

## Discussion

Exercise training is effective in protecting the heart from I/R injury [5]. Given the incompletely defined mechanisms for exercise-induced cardioprotection, particularly for the role of VSMC sarc-K_ATP_ channels, the current study demonstrated a novel pathway of exercise-induced protection through up-regulation of Kir6.1 and shortening of reperfusion recovery in a mouse model of I/R injury.

A number of salient findings are noted in our current study. First of all, exercise training dramatically shortened myocardial tissue oxygenation recovery without affecting oxygenation levels before ischemia or after reperfusion plateaued, indicating a maintained balance between blood flow and oxygen utilization. This result is consistent with the comparable steady-state coronary blood flow from echocardiographic measurements. Given that exercise training improved mitochondrial biogenesis and metabolism [30], the improved oxygenation recovery during the first minutes of reperfusion can only indicate improved recovery of blood flow in the beginning of reperfusion in the hearts of the exercised mice. As a result, improved oxygenation recovery would provide faster delivery of oxygen and nutrients to the myocytes in the area at risk to maintain adequate ATP needed for cell survival within a critical time window when myocytes are at risk. Although exercised and hypertrophied rat hearts showed increased collateral vessel density [31], our echocardiographic measurements of coronary blood flow indicated that there was no significant increase in collateral perfusion in our two-month exercised non-hypertrophied mouse heart model. Therefore, the observed transient improvement in oxygenation recovery must be related to cell signals effective during ischemia or in the very beginning of reperfusion.

**Figure 10:**
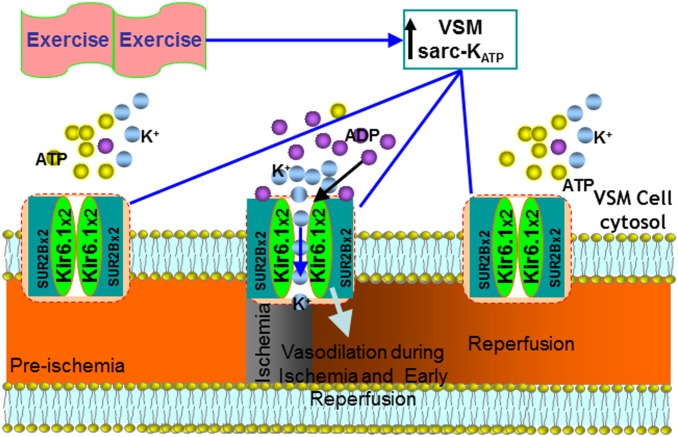
A schematic diagram of our hypothesis: endurance exercise up-regulates Kir6.1 and shortens tissue oxygenation recovery time. During ischemia and at the beginning of reperfusion, the relatively high level of the ADP/ATP opens the VSMC sarc-K_ATP_ channels, leading to hyperpolarization of the VSMC membrane and dilation of the coronary vasculature. Kir6.1-induced transient vasodilation shortens reperfusion recovery time τ and contributes to exercise-induced protection of myocytes from I/R injury.

Secondly, the current study found that exercise training up-regulated Kir6.1, a subunit of VSMC sarc-K_ATP_ channels. Since VSMC sarc-K_ATP_ channels regulate vascular tone and channel activity is determined by the ratio of ADP/ATP, with Mg^2+^ADP activating the channel [17], we rationalize that during ischemia and at the very beginning of reperfusion when heart tissue in the at-risk area is under ischemic stress, ADP/ATP ratio is relatively high, the VSMC sarc-K_ATP_ channels open. Therefore, opening of sarc-K_ATP_ channels during the first minutes of reperfusion may play a critical role in determining oxygenation recovery, as summarized in **Figure 10**. As suggested by our study, Kir6.1 channel was up-regulated following exercise, and myocardial tissue oxygenation recovery time was shortened, as shown in **Figure 1**. Conceivably, any delay of the delivery of oxygen and nutrients to the myocytes at risk due to impaired vascular function and reduced recovery of blood flow may be detrimental to the recovery of myocardial function and may result in further ischemic injury. Therefore, improvements in oxygenation recovery may represent a new mechanism for exercise-induced protection of the ischemic heart. Indeed, inhibition of endogenous VSMC K_ATP_ channels with dominant negative Kir6.1AAA transfection impaired vascular activity, prolonged recovery of myocardial tissue oxygenation, and exacerbated I/R injury. In addition, exercise significantly improved diazoxide-elicited vessel relaxation. These results clearly demonstrated that Kir6.1, constituting the sarc-K_ATP_ channels, played an important role in exercise-induced protection against vascular dysfunction and myocardial I/R injury.

Thirdly, a correlation between myocardial tissue oxygenation recovery time and ischemic time can be drawn. With the introduction of tissue oxygenation recovery time τ, we re-examined our previously published data on recovery of myocardial tissue oxygenation with different ischemic time windows [32]. When ischemic time was extended from 15 to 30 and 60 min, myocardial tissue oxygenation recovery time τ was also extended from 10 to 33 and 50 min, resulting in increased infarct size from 0 to 32.3±0.7% and 45.6±3.8% of AAR. Since myocyte necrosis occurs only hours after the ischemic event, we speculate that slower oxygenation recovery following ischemia may cause a delay in the delivery of oxygen and nutrients to the already stressed myocytes and contribute to I/R injury.

Finally, as previously reported [33]–[35], graded re-oxygenation upon reperfusion with less hyperoxic blood oxygen content but normal blood flow has protective effects on the reperfused tissue against reperfusion injury. Further, post-conditioning with short bouts of ischemia upon reperfusion also protects the reperfused myocardium [32]. However, graded re-oxygenation or post-conditioning is strategically and mechanistically different from Kir6.1AAA-induced prolongation of oxygenation recovery secondary to vascular impairment, which is detrimental to the heart.

A few limitations in the current study are also noted. Due to the lack of a commercially available specific antibody that can discriminate Kir6.1AAA against Kir6.1, Western immunoblotting and co-immunostaining analyses of the efficiency of Ad-Kir6.1AAA-GFP expression in the coronary system was not performed. Secondly, intravenous injection of the adenovirus construct will have a broad range organ distribution including lung, liver, kidney and spleen, which is known to the field. Therefore, we couldn’t exclude any potential adverse effects on the vascular activity from other organs. Lastly, to eventually determine the causative role of Kir6.1 on vascular activity, Kir6.1 knockout and transgenic mouse strains would be the ideal models.

In conclusion, our in vivo EPR oximetry data demonstrated that exercise training up-regulated vascular Kir6.1, shortened myocardial tissue oxygenation recovery time, and reduced I/R injury. These new findings may provide potential targets for pharmacological intervention aiming at VSMC sarc-K_ATP_ channels.
